# P02.26. Integrative care for adolescent mood problems: brief report from a second opinion clinic

**DOI:** 10.1186/1472-6882-12-S1-P82

**Published:** 2012-06-12

**Authors:** K Kemper, B Banasiewicz

**Affiliations:** 1 Wake Forest University Health Sciences, Winston-Salem, USA; 2 University of South Carolina, Columbia, USA

## Purpose

To improve the quality of service and inform care for patients interested in integrative care, we conducted a chart review to describe a) patients whose families had concerns about mood; b) families’ interest in health promotion topics; and c) clinical recommendations.

## Methods

Patients were included if their intake form indicated a concern about mood or depression. We reviewed the comprehensive intake form, physician notes, and laboratory test results. This study was approved by the Wake Forest School of Medicine Institutional Review Board.

## Results

Of the 75 new patients, 34 (45%) noted a concern about mood. The average age was 13 ± 4 years, 68% were female, and patients had an average of 8.6 health concerns such as fatigue, anxiety, headaches, constipation, and pain. Most (88%) received care from other specialists and 71% took medications (average 2.4/patient), most often antidepressants. There was great interest in discussing stress management (86%), nutrition (84%), sleep (82%), and exercise/activity (78%). Most had suboptimal levels of ferritin (65%) and vitamin D (65%). Most parental questions were about fish oil (82%), multivitamins (48%), and minerals (63%).

## Conclusion

Physicians offering integrative care for adolescents with mental health concerns should be prepared to offer advice about healthy lifestyle, particularly stress management, nutrition and nutritional supplements, sleep, and exercise.

